# Supplementary material for: Household transmission of SARS-CoV-2 Omicron variant of concern subvariants BA.1 and BA.2 in Denmark
Source: Nat Commun. 2022 Sep 30;13:5760. doi: 10.1038/s41467-022-33498-0 (PMC9524324; doi:10.1038/s41467-022-33498-0)
Supplement: Supplementary file 1 — Supplementary Information [file 41467_2022_33498_MOESM1_ESM.pdf]

Supplementary Information

Household transmission of SARS-CoV-2 Omicron variant of concern subvariants BA.1 and BA.2 in Denmark

Frederik Plesner Lyngse[1,2,3,*], Carsten Thure Kirkeby[4], Matthew Denwood[4], Lasse Engbo Christiansen[5], Kåre Mølbak[3,4], Camilla Holten Møller[3], Robert Leo Skov[3], Tyra Grove Krause[3], Morten Rasmussen[3], Raphael Niklaus Sieber[3], Thor Bech Johannesen[3], Troels Lillebaek[3,6], Jannik Fonager[3], Anders Fomsgaard[3], Frederik Trier Møller[3], Marc Stegger[3], Maria Overvad[3], Katja Spiess[3], Laust Hvas Mortensen[7,8]

[*]Correspondence to Frederik Plesner Lyngse, fpl@econ.ku.dk. Affiliations: [1]Department of Economics & Center for Economic Behavior and Inequality, University of Copenhagen, Copenhagen, Denmark; [2]Danish Ministry of Health, Copenhagen, Denmark; [3]Statens Serum Institut, Copenhagen, Denmark; [4]Department of Veterinary and Animal Sciences, Faculty of Health and Medical Sciences, University of Copenhagen, Copenhagen, Denmark; [5]Department of Applied Mathematics and Computer Science; Dynamical Systems, Technical University of Denmark, Kgs. Lyngby, Denmark; [6]Global Health Section, University of Copenhagen, Copenhagen, Denmark; [7]Statistics Denmark; [8]Department of Public Health, Faculty of Health and Medical Sciences, University of Copenhagen

S1 Background

This section provides some background characteristics for all of Denmark, i.e., not restricted to the study sample used for the analysis of household transmission.

S1.1 Number of tests

Tables S1 and S2 show the number of antigen (AG) tests and RT-PCR tests in Denmark from 1st December 2021 to 28th February 2022. The tables also provides information on the number of successfully sequenced positive RT-PCR tests by SARS-CoV-2 variant, including their relative proportion. On 20th December 2021, Omicron BA.2 comprised 6% of all infections, and Omicron BA.1 comprised 60%, while Delta comprised 32%. By 5th January 2022, the proportions were 26%, 69%, and 4%, respectively. By 28th January 2022, the proportions were 83%, 16%, and 0%, respectively.

Table S1: Number of tests in Denmark, 1st December 2021—14th January 2022

	AG tests		RT-PCR tests		Variant					
	Tests	Positives	Tests	Positives	Omicron BA.2		Omicron BA.1		Delta	
**Sample date**	**N**	**N**	**N**	**N**	**N**	**%**	**N**	**%**	**N**	**%**
01-12-2021	177,466	1,946	188,055	4,874	0	0	56	2	2,298	98
02-12-2021	217,980	1,908	216,547	4,960	0	0	43	2	2,631	98
03-12-2021	237,466	1,891	189,593	5,599	0	0	33	1	2,343	99
04-12-2021	143,557	1,566	142,053	5,524	0	0	58	2	2,912	98
05-12-2021	144,767	2,133	149,098	5,401	<5	0	101	3	3,330	97
06-12-2021	228,362	2,882	211,715	7,546	0	0	210	5	4,208	95
07-12-2021	233,073	2,778	211,037	7,815	0	0	202	10	1,828	90
08-12-2021	239,457	2,885	207,144	7,088	0	0	198	11	1,541	89
09-12-2021	268,391	2,807	245,061	7,092	0	0	194	15	1,079	85
10-12-2021	274,848	2,596	213,065	7,427	<5	0	203	12	1,465	88
11-12-2021	178,176	2,166	155,858	7,159	0	0	201	17	986	83
12-12-2021	178,068	3,087	167,670	7,669	<5	0	245	21	944	79
13-12-2021	261,270	4,380	232,673	11,221	13	1	398	29	975	70
14-12-2021	254,258	4,529	225,264	12,158	16	2	417	44	503	54
15-12-2021	225,026	4,744	219,481	11,940	14	1	554	48	590	51
16-12-2021	255,308	4,676	258,494	11,317	19	2	516	47	571	51
17-12-2021	273,106	4,550	236,644	11,831	20	4	294	54	228	42
18-12-2021	221,579	4,305	176,426	11,351	32	3	536	56	386	40
19-12-2021	220,878	5,263	182,974	11,637	50	4	687	57	450	37
20-12-2021	248,233	6,379	271,193	15,093	71	6	763	60	412	32
21-12-2021	251,443	6,408	258,469	14,778	66	7	616	65	260	28
22-12-2021	275,256	6,172	272,964	13,584	55	7	525	69	176	23
23-12-2021	277,456	5,037	246,286	14,574	66	8	552	69	177	22
24-12-2021	155,248	4,466	72,576	8,253	24	7	216	63	102	30
25-12-2021	128,702	4,787	73,514	9,163	46	12	282	71	69	17
26-12-2021	168,399	6,072	82,126	12,213	115	15	535	69	126	16
27-12-2021	207,265	7,393	186,654	24,968	353	17	1,482	71	259	12
28-12-2021	213,340	7,253	195,082	24,145	243	16	1,082	72	174	12
29-12-2021	238,567	6,813	216,557	19,208	86	16	393	72	63	12
30-12-2021	314,614	6,387	229,320	21,572	151	16	687	72	107	11
31-12-2021	181,564	4,507	72,610	10,978	95	22	303	69	41	9
01-01-2022	70,576	3,248	75,052	9,821	119	25	333	69	28	6
02-01-2022	224,366	8,394	156,447	22,461	1,086	22	3,483	72	262	5
03-01-2022	239,634	9,284	224,053	28,811	591	25	1,662	69	122	5
04-01-2022	260,020	8,579	207,720	27,044	137	25	391	70	21	4
05-01-2022	225,980	6,023	186,595	20,332	425	26	1,120	69	66	4
06-01-2022	248,176	5,206	212,979	17,996	287	31	611	65	31	3
07-01-2022	240,998	4,777	185,195	16,691	814	35	1,489	63	50	2
08-01-2022	145,731	3,999	136,727	15,682	298	36	497	61	16	2
09-01-2022	204,489	5,890	151,107	18,552	275	40	370	54	21	3
10-01-2022	242,598	6,932	211,923	26,243	787	45	907	52	24	1
11-01-2022	238,728	6,548	197,539	25,647	1,008	47	1,086	51	25	1
12-01-2022	232,379	6,408	190,108	25,505	914	50	881	48	18	1
13-01-2022	255,276	7,167	225,500	26,775	1,122	53	952	45	17	1
14-01-2022	254,397	7,648	207,487	28,937	1,237	57	911	42	10	0

Notes: This table shows the number of antigen (AG) tests and RT-PCR tests in Denmark from 1st December 2021 to 14th January 2022. See Table S2 for 15th January–28th February 2022. The table also provides information on the number of successfully sequenced positive RT-PCR tests by SARS-CoV-2 variant, including their relative proportion.

Table S2: Number of tests in Denmark, 15th January 2022—28th February 2022

	AG tests		RT-PCR tests		Variant					
	Tests	Positives	Tests	Positives	Omicron BA.2		Omicron BA.1		Delta	
Sample date	N	N	N	N	N	%	N	%	N	%
15-01-2022	158,305	6,738	158,450	28,218	995	59	690	41	8	0
16-01-2022	229,720	10,724	175,181	31,224	1,052	62	602	36	8	0
17-01-2022	275,663	11,889	251,475	45,890	1,601	65	843	34	11	0
18-01-2022	267,807	11,957	239,972	44,881	1,740	66	848	32	10	0
19-01-2022	250,180	10,858	228,121	42,231	1,492	68	665	30	5	0
20-01-2022	255,785	10,537	255,930	41,756	1,405	70	602	30	5	0
21-01-2022	259,081	10,631	230,364	41,886	1,855	73	679	27	<5	0
22-01-2022	168,573	9,267	176,584	38,921	1,514	73	531	26	6	0
23-01-2022	219,977	13,546	186,542	42,395	1,750	76	509	22	<5	0
24-01-2022	259,086	14,824	264,291	59,442	2,055	77	548	21	6	0
25-01-2022	224,070	13,180	232,850	54,153	2,110	79	537	20	<5	0
26-01-2022	194,539	11,327	204,754	46,792	1,395	79	351	20	0	0
27-01-2022	186,708	10,245	211,684	43,700	1,354	79	341	20	<5	0
28-01-2022	178,556	9,687	176,774	40,100	996	83	192	16	0	0
29-01-2022	103,985	7,704	117,665	31,452	1,284	85	208	14	0	0
30-01-2022	127,197	11,125	121,152	36,180	2,178	85	341	13	0	0
31-01-2022	170,824	12,542	207,799	58,237	2,459	85	412	14	<5	0
01-02-2022	126,657	9,420	167,812	47,902	756	85	125	14	<5	0
02-02-2022	121,217	9,020	156,780	42,050	754	85	123	14	0	0
03-02-2022	126,582	8,496	174,728	41,304	1,402	91	123	8	0	0
04-02-2022	122,167	8,235	149,397	38,756	1,674	91	148	8	0	0
05-02-2022	68,141	6,517	116,469	34,950	1,822	90	198	10	0	0
06-02-2022	92,761	10,572	135,767	43,725	1,481	90	143	9	0	0
07-02-2022	121,055	12,063	185,996	59,027	1,838	90	168	8	0	0
08-02-2022	115,445	12,405	171,767	57,341	1,885	92	152	7	0	0
09-02-2022	106,750	11,297	164,909	51,999	1,269	93	88	6	0	0
10-02-2022	101,694	9,513	171,175	46,849	1,157	91	91	7	0	0
11-02-2022	90,065	8,164	141,318	40,360	1,788	92	131	7	0	0
12-02-2022	51,769	6,143	101,796	33,899	2,141	93	139	6	0	0
13-02-2022	61,281	7,964	107,830	36,942	2,263	94	122	5	0	0
14-02-2022	64,987	8,168	144,346	46,679	2,657	94	123	4	0	0
15-02-2022	58,753	7,736	129,181	43,404	2,742	95	123	4	<5	0
16-02-2022	54,013	7,022	118,122	37,305	1,435	93	66	4	0	0
17-02-2022	50,999	6,038	125,506	35,433	1,706	95	71	4	0	0
18-02-2022	45,439	5,095	98,501	27,945	2,565	95	102	4	0	0
19-02-2022	33,159	4,332	77,288	26,051	1,452	94	50	3	0	0
20-02-2022	40,950	5,683	90,722	30,837	1,227	95	40	3	0	0
21-02-2022	45,369	5,549	116,929	34,318	2,295	96	75	3	0	0
22-02-2022	39,019	4,696	93,038	28,493	2,142	97	58	3	<5	0
23-02-2022	36,617	4,196	85,397	24,460	1,177	96	43	3	0	0
24-02-2022	34,108	3,498	88,565	21,513	1,787	96	49	3	<5	0
25-02-2022	31,432	3,126	67,992	17,441	2,062	97	48	2	0	0
26-02-2022	21,121	2,565	47,127	14,626	2,343	97	54	2	<5	0
27-02-2022	23,811	3,076	56,814	17,621	1,887	97	40	2	<5	0
28-02-2022	27,060	3,260	82,244	22,349	1,621	97	35	2	<5	0

Notes: This tables shows the number of antigen (AG) tests and RT-PCR tests in Denmark from 15th January to 28th February 2022. See Table S1 for 1st December 2021 to 14th January 2022. The table also provides information on the number of successfully sequenced positive RT-PCR tests by SARS-CoV-2 variant, including their relative proportion.

S1.2 Sample selection to WGS

In Denmark, individuals can be tested in the community track (TestCenter Denmark) or in the healthcare track (Hospitals), which includes hospitalized patients, nursing home residents, and healthcare personnel (see Schønning et al.[20] for elaboration). Only a proportion of all positive RT-PCR tests were sampled for whole genome sequencing (WGS). Both TestCenter Denmark and Hospitals sample positive RT-PCR tests randomly for WGS. However, all hospitalized patients were tested for SARS-CoV-2 and all positive tests were subject to WGS for treatment purposes. Figure S1 shows the sampling probability for WGS within the study period by TestCenter Denmark and Hospitals. Panel a and b shows the sampling probability by age. For positive RT-PCR tests at TestCenter Denmark, there was no selection bias on age, whereas in hospitals, there was an increased sampling probability by age for older individuals. Panel c shows the sampling probability for WGS by sample Ct value for TestCenter Denmark (we only obtained Ct values from TestCenter Denmark). There was no sampling bias for Ct values <35. The probability of obtaining a successfully sequenced genome was correlated with the sample Ct value.

Figure S1: WGS sampling probability of positive RT-PCR tests

By age

(a) TestCenter Denmark

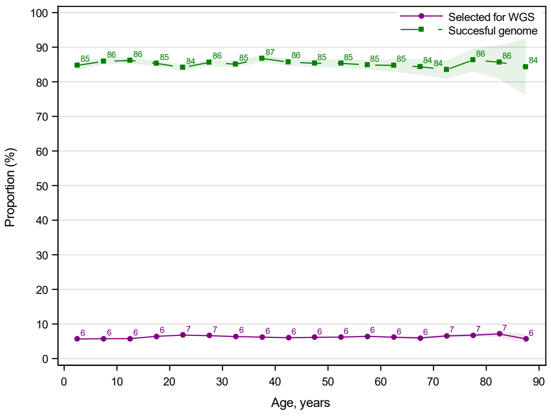

(b) Hospitals

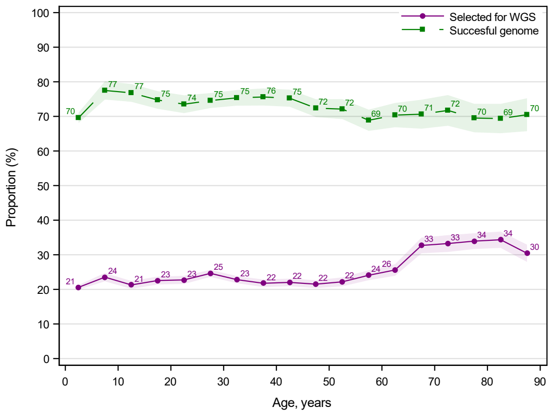

By Ct value

(c) TestCenter Denmark

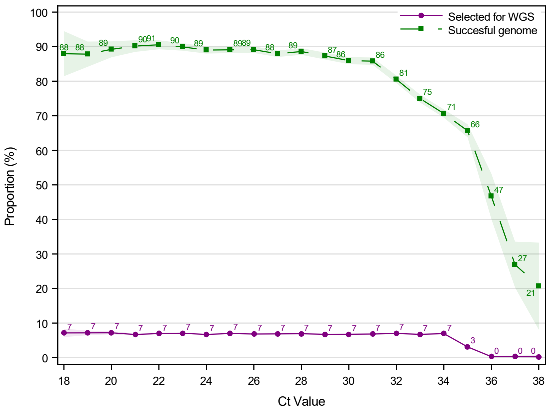

Notes: This figure shows the sampling probability (purple) of positive RT-PCR tests for WGS by testing place (TestCenter Denmark and Hospitals). The figure also show the proportion of the sampled test that had a successfully sequenced genome (green). Only Ct values from TestCenter Denmark were available. The markers show the point estimates of the mean. The shaded areas show the 95% confidence bands.

S2 Descriptive analyses

S2.1 Summary statistics

In this section, we present additional summary statistics.

Table S3 presents the same as Table 1 in proportions (%), i.e., summary statistics for primary cases and household contacts separately. Tables S5 and S4 presents the summary statistics stratified by the characteristics of the primary case. Thus, the SAR presented in Tables 1 and S3 refer to the characteristics of the contacts, whereas the SAR presented in Tables S4 and S5 refer to the characteristics of the primary cases.

Table S3: Summary statistics (primary cases and contacts reported separately)

	Omicron - BA.2				Omicron - BA.1			
	Primary Cases	Household Contacts	Secondary Cases	SAR (%)	Primary Cases	Household Contacts	Secondary Cases	SAR (%)
**Total, N**	11,348	25,859	10,102	39	11,330	24,729	7,217	29
**Sex, %**								
Male	49	50	47	37	48	50	48	28
Female	51	50	53	42	52	50	52	31
**Age, %**								
0-9 years	18	19	19	40	10	19	20	32
10-19 years	29	17	15	33	26	18	17	27
20-29 years	16	10	10	38	22	13	13	29
30-39 years	12	16	22	54	15	13	18	42
40-49 years	9	22	21	38	11	20	18	27
50-59 years	9	11	9	31	10	12	9	21
60-69 years	4	3	3	34	5	4	3	25
70+ years	2	2	1	35	3	2	2	28
**Household size, %**								
2 persons	32	14	15	42	36	17	18	31
3 persons	24	21	19	37	24	22	21	27
4 persons	30	40	41	41	27	37	39	31
5 persons	11	20	20	38	11	19	18	28
6 persons	2	5	5	33	2	5	4	25
**Vaccination status, %**								
Unvaccinated[a]	29	26	28	42	22	27	31	34
Fully vaccinated[b]	41	31	33	41	52	38	41	31
Booster vaccinated	30	43	39	36	26	35	28	24

Notes: Summary statistics for primary cases are shown separately from summary statistics for household contacts, secondary cases and SAR. Thus the SAR reflects the proportion of household contacts that tested positive, irrespective of the characteristics of the primary case. The raw numbers (N) for each category is presented in appendix Table 1. Summary statistics stratified by the primary case level are presented in Tables S4 and S5. [a]Unvaccinated includes individuals with partial vaccination (24 primary cases and 18 contacts). [b]Fully vaccinated includes unvaccinated individuals with previous infection.

Table S4: Summary Statistics, stratified by primary case level

	Omicron - BA.2				Omicron - BA.1			
	**Primary Cases**	**Household Contacts**	**Secondary Cases**	**SAR (%)**	**Primary Cases**	**Household Contacts**	**Secondary Cases**	**SAR (%)**
**Total, N**	11,348	25,859	10,102	39	11,330	24,729	7,217	29
**Sex of primary case, N**								
Male	5,504	12,771	5,145	40	5,487	12,090	3,558	29
Female	5,844	13,088	4,957	38	5,843	12,639	3,659	29
**Age of primary case, N**								
0-9 years	2,018	5,745	3,206	56	1,123	3,176	1,297	41
10-19 years	3,287	8,793	2,567	29	2,909	7,682	1,526	20
20-29 years	1,788	3,081	971	32	2,496	4,251	1,018	24
30-39 years	1,406	3,355	1,387	41	1,646	3,907	1,466	38
40-49 years	1,078	2,521	1,008	40	1,254	3,024	1,025	34
50-59 years	1,015	1,495	590	39	1,082	1,739	543	31
60-69 years	477	571	240	42	520	629	217	34
70+ years	279	298	133	45	300	321	125	39
**Household size of primary case, N**								
2 persons	3,675	3,675	1,529	42	4,087	4,087	1,278	31
3 persons	2,674	5,348	1,961	37	2,756	5,512	1,491	27
4 persons	3,438	10,314	4,180	41	3,053	9,159	2,830	31
5 persons	1,283	5,132	1,975	38	1,199	4,796	1,329	28
6 persons	278	1,390	457	33	235	1,175	289	25
**Vaccination status of primary case, N**								
Unvaccinated[a]	3,285	8,582	4,232	49	2,497	6,231	2,186	35
Fully vaccinated[b]	4,667	11,179	3,695	33	5,844	13,200	3,594	27
Booster vaccinated	3,396	6,098	2,175	36	2,989	5,298	1,437	27

Notes: Summary statistics are stratified by the primary case level. For example, there were 2,018 primary cases aged 0-9 years with Omicron BA.2 living with 5,745 household contacts, of which 3,206 tested positive, yielding a SAR of 56%. Thus the SAR reflects the proportion of household contacts that tested positive, conditional of the characteristics of the primary case. The proportions (%) for each category is presented in Table S5. See Tables 1 and S3 for contacts and secondary cases grouped by their own characteristics. [a]Unvaccinated includes individuals with partial vaccination (24 primary cases and 18 contacts). [b]Fully vaccinated includes unvaccinated individuals with previous infection.

Table S5: Summary Statistics, stratified by primary case level

	Omicron - BA.2				Omicron - BA.1			
	Primary Cases	Household Contacts	Secondary Cases	SAR (%)	Primary Cases	Household Contacts	Secondary Cases	SAR (%)
**Total, N**	11,348	25,859	10,102	39	11,330	24,729	7,217	29
**Sex of primary case, %**								
Male	49	49	51	40	48	49	49	29
Female	51	51	49	38	52	51	51	29
**Age of primary case, %**								
0-9 years	18	22	32	56	10	13	18	41
10-19 years	29	34	25	29	26	31	21	20
20-29 years	16	12	10	32	22	17	14	24
30-39 years	12	13	14	41	15	16	20	38
40-49 years	9	10	10	40	11	12	14	34
50-59 years	9	6	6	39	10	7	8	31
60-69 years	4	2	2	42	5	3	3	34
70+ years	2	1	1	45	3	1	2	39
**Household size of primary case, %**								
2 persons	32	14	15	42	36	17	18	31
3 persons	24	21	19	37	24	22	21	27
4 persons	30	40	41	41	27	37	39	31
5 persons	11	20	20	38	11	19	18	28
6 persons	2	5	5	33	2	5	4	25
**Vaccination status of primary case, %**								
Unvaccinated[a]	29	33	42	49	22	25	30	35
Fully vaccinated[b]	41	43	37	33	52	53	50	27
Booster vaccinated	30	24	22	36	26	21	20	27

Notes: Summary statistics are stratified by the primary case level. Thus the SAR reflects the proportion of household contacts that tested positive, conditional on the characteristics of the primary case. The raw numbers (N) for each category is presented in appendix Table S4. The secondary attack rate (SAR) refers to the characteristics of the primary cases. Household contacts and secondary cases are grouped based on the primary case characteristics. See Tables 1 and S3 for contacts and secondary cases grouped by their own characteristics. [a]Unvaccinated includes individuals with partial vaccination (24 primary cases and 18 contacts). [b]Fully vaccinated includes unvaccinated individuals with previous infection.

S2.2 Testing dynamics, 14-day follow-up

In this section, we present evidence of the testing dynamics over a 14-day follow-up period, rather than a 7-day period as in the main manuscript. Figure S2 presents the probability of being tested and testing positive over a 14-day follow-up, similar to the 7-day follow-up period used in Figure 1. Figure S3 presents the 14-day SAR for households infected with the Omicron BA.1, BA.2, and Delta VOC, as well as those without an identified variant.

Figure S2: Probability of being tested and testing positive, 14-day follow-up

(a) Probability of testing (b) Probability of testing positive

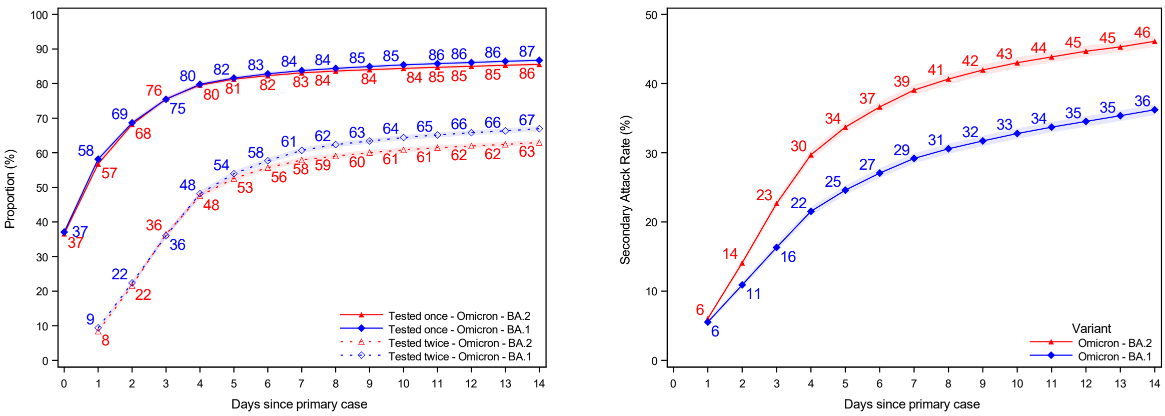

Notes: Panel (a) shows the probability of potential secondary cases being tested after a primary case has been identified within the household. Panel (b) shows the probability of potential secondary cases that test positive subsequently to a primary case being identified within the household. Note that the latter is not conditional on being tested, i.e., the denominator contains test negative individuals and untested individuals. The x-axes shows the days since the primary case tested positive, and the y-axes shows the proportion of individuals either being tested (a) or testing positive (b) with either antigen or RT-PCR tests, based on the subvariant of the primary case. The SAR for each day relative to the primary case can be read directly from panel (b). For example, the SAR on day 7 is 39% for BA.2 (red) and 29% for BA.1 (blue), whereas the SAR on day 14 is 46% and 36%, respectively. The markers show the point estimates of the mean. The shaded areas show the 95% confidence bands with cluster-robust standard errors at the household level. Appendix Figure S3 also presents the 14-day SAR for the Delta VOC and those without a known variant.

Figure S3: Probability of testing positive, 14-day follow-up

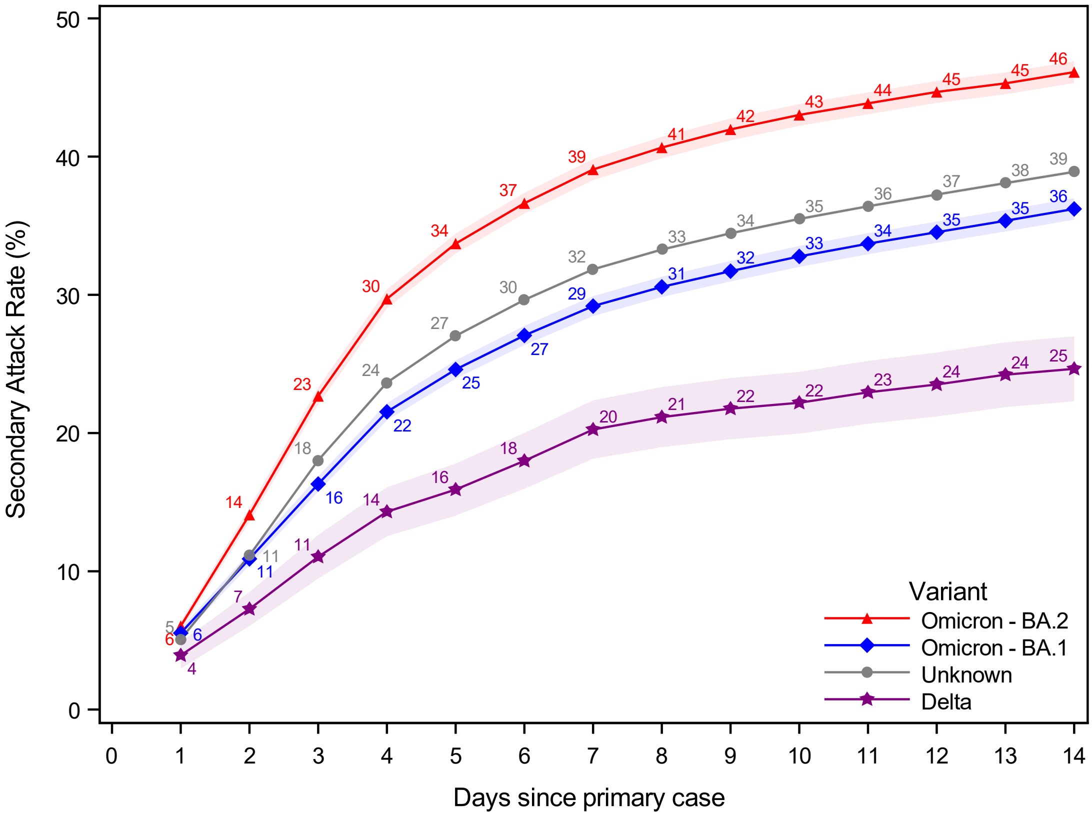

Notes: This figure shows the probability of potential secondary cases that test positive subsequently to a primary case being identified within the household in a 14-day follow-up period. Note that the latter is not conditional on being tested, i.e., the denominator contains test negative individuals and untested individuals. The x-axes shows the days since the primary case tested positive, and the y-axes shows the proportion of individuals testing positive with either antigen or RT-PCR tests, based on the subvariant of the primary case. The SAR for each day relative to the primary case can be read directly from the figure. For example, the SAR on day 14 is 46% for Omicron BA.2 (red), 36% for BA.1 (blue), 39% for those without a known variant (gray), and 25% for Delta (purple). The markers show the point estimates of the mean. The shaded areas show the 95% confidence bands with cluster-robust standard errors at the household level.

S2.3 Viral load of primary cases

This section provides descriptive statistics on the viral load of the primary case samples. Figure S4 shows the density plots of sample Ct values for primary cases infected with Omicron BA.1 and BA.2 stratified by their vaccination status. The distributional values are presented in Table S6. Primary cases infected with Omicron BA.2 had a slightly higher sample viral load (lower Ct value).

Figure S4: Ct values of primary cases

(a) Unvaccinated (b) Fully vaccinated (c) Booster- vaccinated

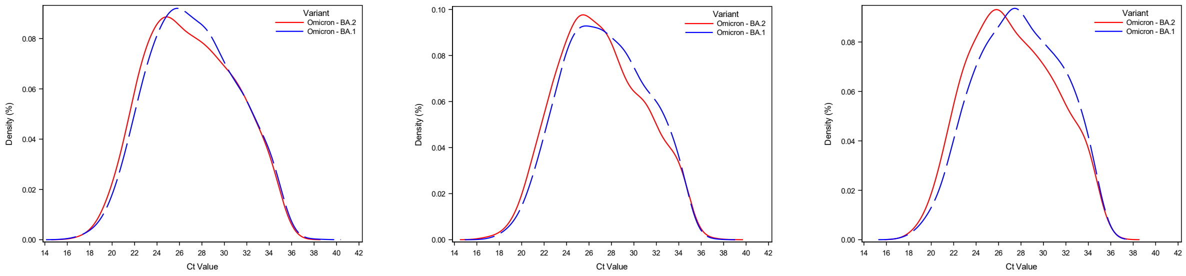

Notes: This figure shows the density plots for primary cases infected with Omicron BA.1 (blue) and BA.2 (red) stratified by their vaccination status.

Table S6: Ct values of primary cases

Vaccination status	Subvariant	Q1	Median	Q3	Mean	STD	N
Booster vaccinated	Omicron - BA.1	25.08	27.74	30.71	27.80	3.69	2,989
	Omicron - BA.2	24.30	26.94	30.14	27.21	3.80	3,396
	Difference / STD	-0.21	-0.20	-0.15	-0.15		
Fully vaccinated	Omicron - BA.1	24.58	27.28	30.25	27.42	3.70	5,844
	Omicron - BA.2	24.17	26.73	29.87	27.01	3.78	4,667
	Difference / STD	-0.11	-0.14	-0.10	-0.10		
Not vaccinated	Omicron - BA.1	24.37	27.13	30.13	27.28	3.81	2,497
	Omicron - BA.2	24.02	26.84	30.14	27.03	3.89	3,285
	Difference / STD	-0.09	-0.07	0.00	-0.06		

Notes: This table provides distributional values for the Ct values of primary case samples. "Difference / STD" denotes the difference of primary cases with BA.2 and BA.1 relative to the standard deviation of BA.1 primary cases, within vaccination group.

S3 Alternative presentation of main results

S3.1 Contrasts

In this section, we present some of our main estimates in an alternative way, showing the estimates for comparison across different vaccination groups.

Figure S5 shows a full comparison of our main estimates across vaccination groups with different reference groups. We can see the relative effect of vaccination dependent on their vaccination status by choosing the *Contrast* (column) and compare their *infectiousness* to the vaccination status of a similar primary case by choosing the *Reference* (row). For example, unvaccinated primary cases (Contrast=Unvaccinated) compared to fully vaccinated primary cases (Reference=Fully vaccinated) have an *infectiousness* of OR=0.98 when infected with BA.1 (blue) and OR=1.19 when infected with BA.2 (red), (also presented in Table 3). The interaction effect (black) is the relative effect of BA.2 and BA.1 (BA.2/BA.1, 0.98/1.19=1.21). This interaction term can be interpreted as the additional OR associated with BA.2 (relative to BA.1) within the comparison. If we switch the comparison group to comparing unvaccinated primary cases (Contrast=Unvaccinated) to booster-vaccinated primary cases (Contrast=Booster vaccinated), we have an OR=1.19 when infected with BA.1 and an OR=1.39 when infected with BA.2, which indeed is higher than when the comparison group is fully vaccinated primary cases, as expected. In a similar way, when we compare fully vaccinated individuals (Contrast=Fully vaccinated) with unvaccinated individuals, we compare with someone with less immunity, whereas, when we compare them with and booster-vaccinated individuals, we compare with someone with more immunity. Thus, we expect the OR estimate to flip from below one to above one. The estimates for *Susceptibility* to infection is read in a similar way, but for household contacts. Lastly, the *Combined* effect of vaccination shows the effect of both the primary case and household contact having the same vaccination status. Thus, for households infected with BA.1, the OR=1.25 if both the primary case and household are unvaccinated (Contrast=Unvaccinated) compared to when both are fully vaccinated (Reference=Fully vaccinated).

Figure S5: Effect of vaccination, contrast plot

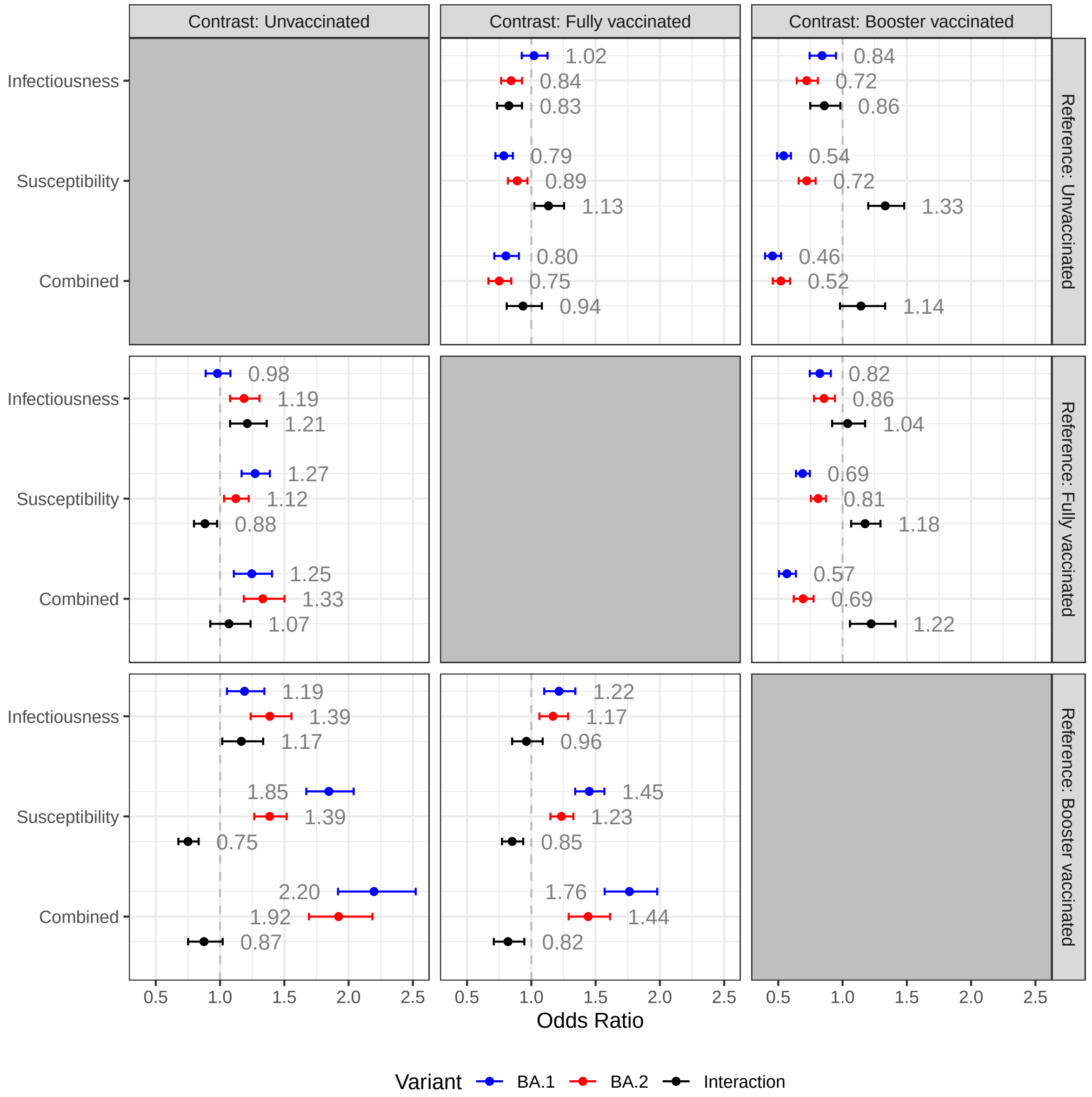

Notes: This figure shows a full comparison of our main estimates across vaccination groups with different reference groups. We can see the relative effect of vaccination dependent on their vaccination status by choosing the *Contrast* (column) and compare their *infectiousness* to the vaccination status of a similar primary case by choosing the *Reference* (row). For example, unvaccinated primary cases (Contrast=Unvaccinated) compared to fully vaccinated primary cases (Reference=Fully vaccinated) have an *infectiousness* of OR=0.98 when infected with BA.1 (blue) and OR=1.19 when infected with BA.2 (red), (also presented in Table 3). The interaction effect (black) is the relative effect of BA.2 and BA.1 (BA.2/BA.1, 0.98/1.19=1.21). This interaction term can be interpreted as the additional OR associated with BA.2 (relative to BA.1) within the comparison. Note that the top/right subplots are simply the inverse of the lower/left subplots. The markers show the point estimates of the mean. The horizontal bars show the 95% confidence bands with cluster-robust standard errors at the household level.

Table S7 shows the OR for infection with BA.2 compared to BA.1 for each combination of vaccination status of both the primary case and household contact. For example, when both the primary case and the household contact are unvaccinated the OR=1.60, and when the primary case is unvaccinated and the contact is booster-vaccinated the OR=2.14. The relative transmission of BA.2 compared to BA.1 is larger than one across all combinations of vaccination groups. The relative transmission of BA.2 compared to BA.1 is generally higher for unvaccinated primary cases across all vaccination groups of household contacts.

Table S7: OR estimates of BA.2 compared to BA.1 by vaccination status

	**Primary case**		
	**Unvaccinated**	**Fully vaccinated**	**Booster vaccinated**
**Household contact**			
Unvaccinated	1.60	1.32	1.38
	(1.44-1.79)	(1.19-1.47)	(1.21-1.57)
Fully vaccinated	1.82	1.50	1.56
	(1.63-2.03)	(1.37-1.64)	(1.38-1.76)
Booster vaccinated	2.14	1.76	1.83
	(1.91-2.38)	(1.60-1.94)	(1.65-2.04)

Notes: This table shows the OR for BA.2 compared to BA.1 for each combination of vaccination status of both the primary case and household contact. 95%-confidence intervals are shown in parentheses with cluster-robust standard errors at the household level.

Table S8 presents the model estimates (similar to Table S12, model I) with interaction terms instead of a full specification of contrasts. We present both the estimates as dummy coded (column I) and effect coded (column II). The odds ratio estimates (column III) can be calculated as the exponentiation of the dummy coded estimates, e.g., exp(-1.21)=0.30.

Table S8: Model estimates

	I		II		III	
	**Dummy coded**		**Effect coded**		**Odds ratio**	
	**Estimate**	**(95%-CI)**	**Estimate**	**(95%-CI)**	**OR**	**(95%-CI)**
Intercept	-1.21	(-1.55;-0.87)	-0.61	(-0.67;-0.55)	0.30	(0.21-0.42)
**Variant**						
Omicron BA.1	ref	(.)	ref	(.)	ref	(.)
Omicron BA.2	0.41	(0.31;0.50)	0.25	(0.22;0.28)	1.50	(1.37-1.64)
**Household contact, vaccination status**						
Booster vaccinated	-0.37	(-0.45;-0.29)	-0.25	(-0.29;-0.21)	0.69	(0.64-0.75)
Fully vaccinated	ref	(.)	ref	(.)	ref	(.)
Unvaccinated	0.24	(0.15;0.33)	0.22	(0.17;0.26)	1.27	(1.17-1.39)
**Primary case, vaccination status**						
Booster vaccinated	-0.19	(-0.29;-0.10)	-0.14	(-0.19;-0.09)	0.82	(0.75-0.91)
Fully vaccinated	ref	(.)	ref	(.)	ref	(.)
Unvaccinated	-0.02	(-0.12;0.08)	0.11	(0.06;0.16)	0.98	(0.89-1.08)
**Household contact, interaction**						
BA.2 X Booster vaccinated	0.16	(0.07;0.26)	0.07	(0.05;0.10)	1.18	(1.07-1.29)
BA.2 X Fully vaccinated	ref	(.)	ref	(.)	ref	(.)
BA.2 X Unvaccinated	-0.12	(-0.23;-0.02)	-0.07	(-0.10;-0.04)	0.88	(0.80-0.98)
**Primary case, interaction**						
BA.2 X Booster vaccinated	0.04	(-0.08;0.16)	-0.02	(-0.06;0.02)	1.04	(0.92-1.18)
BA.2 X Fully vaccinated	ref	(.)	ref	(.)	ref	(.)
BA.2 X Unvaccinated	0.19	(0.08;0.31)	0.06	(0.02;0.09)	1.21	(1.08-1.36)
**Primary case, age**						
0-9 years	0.79	(0.68;0.89)	0.21	(0.12;0.31)	2.19	(1.97-2.44)
10-19 years	-0.06	(-0.14;0.03)	-0.63	(-0.70;-0.56)	0.94	(0.87-1.03)
20-29 years	ref	(.)	ref	(.)	ref	(.)
30-39 years	0.54	(0.45;0.63)	-0.03	(-0.11;0.04)	1.71	(1.56-1.88)
40-49 years	0.60	(0.49;0.70)	0.03	(-0.05;0.10)	1.82	(1.64-2.02)
50-59 years	0.62	(0.51;0.73)	0.05	(-0.04;0.14)	1.86	(1.66-2.09)
60-69 years	0.87	(0.70;1.04)	0.30	(0.17;0.43)	2.39	(2.02-2.83)
70+ years	1.22	(0.96;1.47)	0.65	(0.43;0.86)	3.38	(2.62-4.35)
**Household contact, age**						
0-9 years	-0.30	(-0.40;-0.21)	-0.18	(-0.26;-0.11)	0.74	(0.67-0.81)
10-19 years	-0.21	(-0.30;-0.13)	-0.09	(-0.15;-0.03)	0.81	(0.74-0.88)
20-29 years	ref	(.)	ref	(.)	ref	(.)
30-39 years	0.37	(0.28;0.45)	0.49	(0.43;0.55)	1.44	(1.33-1.57)
40-49 years	0.11	(0.03;0.20)	0.23	(0.18;0.29)	1.12	(1.03-1.22)
50-59 years	-0.13	(-0.23;-0.04)	-0.01	(-0.08;0.05)	0.87	(0.80-0.96)
60-69 years	-0.29	(-0.43;-0.15)	-0.17	(-0.27;-0.06)	0.75	(0.65-0.86)
70+ years	-0.50	(-0.71;-0.29)	-0.38	(-0.56;-0.21)	0.61	(0.49-0.75)
**Household size**						
2 persons	0.14	(0.06;0.21)	0.21	(0.15;0.27)	1.15	(1.07-1.23)
3 persons	-0.09	(-0.15;-0.02)	-0.01	(-0.06;0.04)	0.92	(0.86-0.98)
4 persons	ref	(.)	ref	(.)	ref	(.)
5 persons	-0.10	(-0.18;-0.03)	-0.03	(-0.09;0.03)	0.90	(0.84-0.97)
6 persons	-0.32	(-0.45;-0.18)	-0.24	(-0.35;-0.14)	0.73	(0.64-0.84)
**Household contact, sex**						
Male	ref	(.)	ref	(.)	ref	ref
Female	0.16	(0.13;0.20)	0.16	(0.13;0.20)	1.18	(1.14-1.22)
**Primary case, sex**						
Male	ref	(.)	ref	(.)	ref	(.)
Female	-0.02	(-0.07;0.03)	-0.02	(-0.07;0.03)	0.98	(0.93-1.03)
**Primary case sample date**						
Fixed effects - Not shown	-	-	-	-	-	
Number of observations	50,588		50,588		50,588	
Number of households	22,678		22,678		22,678	

Notes: This table provides model estimates of our main specification (model 1) with an interaction effect. 95% confidence intervals are shown in parentheses. Standard errors are clustered on the household level. To facilitate the interpretation of the main effects, the estimates are presented using dummy coding (I) and effect coding (II), as well as the corresponding odds ratio estimates (III). The odds ratio estimates are also presented in the appendix Table S12, column I.

S4 Robustness

This section provides several robustness checks to support our main analysis. We present measures of the goodness of model fit in appendix section S4.1. In appendix section S4.2, we investigate the potential misclassification of cases. Odds ratio estimates for the main model is presented in Table S12, model 1. In appendix section S4.3, we investigate the robustness of our main results by running the same regression model to specific strata of the data.

S4.1 Model selection

Table S9 provides measures of model fit using the Akaike Information Criterion (AIC) across 4 different model specifications.

Table S9: Model selection

**Model**	**I**	**II**	**III**	**IV**
AIC (Akaike Information Criterion)	61,585	61,599	61,614	61,623
Omicron BA.2 household	YES	YES	YES	YES
Household contact vaccination status	YES	YES	YES	YES
Primary case vaccination status	YES	YES	YES	YES
Household contact vaccination status X Omicron BA.2 household	YES	YES	NO	NO
Primary case vaccination status X Omicron BA.2 household	YES	NO	YES	NO
Primary case age	YES	YES	YES	YES
Household contact age	YES	YES	YES	YES
Household size	YES	YES	YES	YES
Primary case sex	YES	YES	YES	YES
Household contact sex	YES	YES	YES	YES
Primary case sample date	YES	YES	YES	YES
Number of observations	50,588	50,588	50,588	50,588
Number of households	22,678	22,678	22,678	22,678

Notes: This table provides estimates of the goodness of fit for the model. Model I includes an interaction with Omicron BA.2 both for susceptibility and infectiousness, and is the one used in the study. Model II includes an interaction with Omicron BA.2 only for susceptibility. Model III includes an interaction with Omicron BA.2 only for infectiousness. Model IV includes neither an interaction with Omicron BA.2 only for susceptibility nor infectiousness.

S4.2 Misclassification of cases

One of the main potential weaknesses of our empirical approach is the assumption that primary and secondary cases are classified correctly, i.e., that the assumed household transmission did in fact occur from primary to secondary cases within the household.

There are three overall concerns with misclassifications: i) Misclassification of tertiary cases as secondary cases; ii) Misclassification of primary cases iii) Secondary cases are identified as being infected in the household, but are in fact infected by the outside community. Below, we separately address each of the three concerns.

i) Misclassification of tertiary cases as secondary cases

Tertiary cases could in theory be misclassified as secondary cases. In this study, this should not impose an issue when comparing transmission across subvariants, as long as the misclassification is the same across subvariants. However, if for example one subvariant has a shorter serial interval time, this could lead to a difference in the number of tertiary cases that is correlated with the household subvariant. To address this potential issue, we used two-person households as a validation measure, as they, by definition, do not have any tertiary household cases. First, we compared the SAR over time for multi-person households (Figure S6, panel a) and two-person households (panel b). The 14-day SAR was relatively similar across the two panels.

Figure S6: Probability of testing positive, stratified by household size

(a) All households

(b) 2-person households

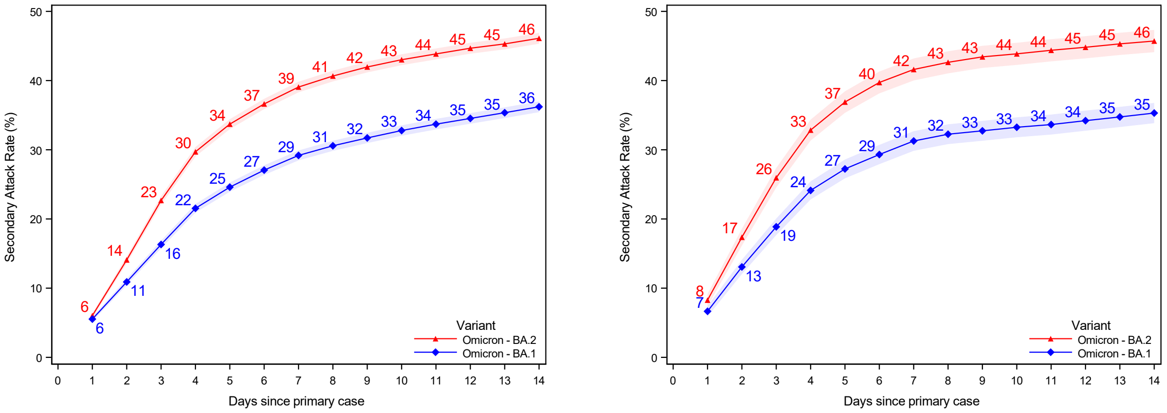

Notes: Panel (a) shows the same as Figure S2.b, i.e., the probability of household contacts testing positive after a primary case has been identified within the household for households with 2-6 members. Panel (b) shows the same estimates stratified by 2-person households. The x-axes show the days since the primary case tested positive, and the y-axes show the proportion of individuals either being tested (a) or testing positive (b) with either antigen or RT-PCR tests, stratified for the subvariant of the primary case. The SAR for each day according to the subvariant primary case can be read directly from the two panels. The markers show the point estimates of the mean. The shaded areas show the 95% confidence bands with cluster-robust standard errors at the household level.

Next, we used the SAR estimates from Figure S6 to calculate the relative SAR of Omicron BA.2 and BA.1 households for multi-person and two-person households separately for each day. If tertiary cases were more prevalent for one subvariant, we would expect the ratio for multi-person households to diverge from the ratio for two-person households over time. We found similar ratios across the two subvariants, suggesting no differential misclassification of tertiary cases across subvariants, and thus not a major limitation in our study (Table S10).

Table S10: Relative SAR over time since primary case by household size and day since primary case

	SAR				Relative SAR	
	All households		2-person households		All households	2-person households
Day	BA.1	BA.2	BA.1	BA.2	(BA.2/BA.1)	(BA.2/BA.1)
1	6	6	7	8	1.0	1.1
2	11	14	13	17	1.3	1.3
3	16	23	19	26	1.4	1.4
4	22	30	24	33	1.4	1.4
5	25	34	27	37	1.4	1.4
6	27	37	29	40	1.4	1.4
7	29	39	31	42	1.3	1.4
8	31	41	32	43	1.3	1.3
9	32	42	33	43	1.3	1.3
10	33	43	33	44	1.3	1.3
11	34	44	34	44	1.3	1.3
12	35	45	34	45	1.3	1.3
13	35	45	35	45	1.3	1.3
14	36	46	35	46	1.3	1.3

Notes: The SAR estimates are presented in Figure S6.

ii) Misclassification of primary cases

Identifying the correct primary cases within the households is important for our study, because this determines if the household is counted as infected with Omicron BA.1 or BA.2 and is used to estimate the infectiousness. In theory, the first identified case (i.e., the index case) may not be the primary case of a household transmission chain. In our study, we classify cases based on the timing of the tests and test results. This could pose a problem, if for example vaccination status and/or symptoms is correlated with the propensity of being tested or if individuals in general have a low test propensity. The

ideal setting would be to have test results on all household members on, e.g., a daily basis, to make sure that secondary cases in fact test positive after the primary case. During our study period, Denmark had a relatively high test capacity and test intensity, with approximately 10% of the population being tested each day (Tables S1 and S2) and a large proportion of household contacts being tested several times after the primary case (Figure 1). We can classify the household contacts into five types based on their observed tests and test results within the 7 days of exposure:

Type	Test 1	Test 2	Potential primary case
A	None	None	Yes
B	Positive	None	Yes
C	Negative	None	No
D	Negative	Positive	No
E	Negative	Negative	No

- Type A can potentially be the primary case, because they do not have any test results.
- Type B can potentially be the primary case, but just identified after the index case.
- Type C, D and E cannot be the primary case, because they have a negative test after the the index case.

Using the classification from above, we can restrict our analysis sample to only include households, where all contacts have a negative test result after the primary case, i.e., only including households comprising types C, D, and E. For these households, we assume no misclassification of primary cases (assuming a high test sensitivity). This leaves us with a sub-sample of 59% of all households and 54% of all contacts. We estimate our regression model using this sub-sample (Table S13, model V). Furthermore, we also estimate our regression model, only including secondary cases found on day 2-7 and 3-7 (Table S13, model VI and VII). The results are qualitatively similar to the main results presented in the paper, supporting the robustness of our conclusions.

iii) Misclassification of community cases as secondary household cases

We assume that all household contacts that test positive within 7 days of the primary case were infected by the household primary case. In theory, these secondary cases could be infected by the outside community and thus be misclassified as secondary household cases. We addressed this potential concern of misclassification in several steps. First, we estimated the probability that the secondary cases were in fact infected with the same variant as the primary case. Of our 17,319 secondary cases, 1,567 (9%) had a successfully sequenced genome from their sample. (Due to the high number of cases at the time of our study, the public health authorities could not WGS all positive RT-PCR test samples.) Nevertheless, using this sub-sample, we found that 96% of all secondary cases were infected with the same variant as the primary case (Table S11, specification I). When we split the sample based on the variant of the primary case, we found similar estimates (specification II and III).

These estimates are a necessary, but not sufficient, condition to rule out misclassification due to community infection. For example, if the local community is mainly infected with one subvariant and that is the same subvariants as within the household, we would not be able to distinguish secondary cases infected by the local community from those infected within the household using these estimates. However, if we focus on households infected with a subvariant that is different from the circulating subvariants in the local community, we can gauge our estimate of community infections. To do so, we calculated the overall proportion of BA.1 and BA.2 cases within each municipality on a weekly basis to allow for variation across both time and space. Thus, we can now identify households infected with BA.2 that are located in a community that is predominately infected with other subvariants (Delta and BA.1). We focus on municipalities, where less than 50% of all cases are the same subvariant as the household. If secondary cases were infected by the community, we would expect to find a lower probability of them having the same variant as the primary case, whereas, we would expect to find a high probability, if they were infected by the household. We found that in households located in municipalities with predominantly other variants, secondary cases have a slightly lower probability of being

infected with the same variant as the primary case (93%, specification IV). However, the misclassification is modest. Lastly, as the probability of being infected in the community is correlated with the community incidence, we split municipalities based on their overall incidence. Again, we find similar estimates (specification VI and VII).

Overall, we interpret these results as a low possibility of misclassification of secondary cases due to community infection.

Table S11: Probability of secondary cases having the same variant as the primary case

Specification	I	II	III	IV	V	VI	VII
Municipalities				Share of same variant		Incidence	
				Below 50%	Above 50%	Above median	Below median
Primary case variant	All	BA.1	BA.2	All	All	All	All
Prob same variant	0.96	0.94	0.97	0.93	0.97	0.95	0.98
(95%-CI)	(0.95-0.97)	(0.92-0.96)	(0.96-0.98)	(0.90-0.96)	(0.96-0.98)	(0.93-0.97)	(0.97-0.99)
Number of observations	1,567	656	911	462	1,105	1,114	453
Number of households	1,338	570	768	394	944	931	407
Number of municipalities	93	88	87	76	92	84	87

Notes: 95%-confidence intervals are shown in parentheses with cluster-robust standard errors at the household level.

S4.3 Robustness of main results

We investigated the robustness of our main results by running the same regression model to specific strata of the data. The estimates are shown in Tables S12-S16, where columns I-XIV refer to the following:

I, Main) The analysis presented in the main manuscript for reference.

II, 14 day SAR) Using a 14-day follow-up instead of a 7-day follow-up.

III, 2-person households) Only including households with two persons to account for the natural weighting bias from different sizes of households.

IV, Primary cases >10 years) Excluding households, where the primary case was below 10 years.

V, Only tested negative) Restricting the sample to only include households, where all contacts had tested negative after subsequent to the primary case.

VI, Only cases on day 2-7) Only including secondary cases identified on day 2-7 instead of 1-7.

VII, Only cases on day 3-7) Only including secondary cases identified on day 3-7 instead of 1-7.

VIII, 20DEC-04JAN) Only including households, where the primary case tested positive during the holidays, 20th December 2021 to 4th January 2022, rather than the full period (20th December 2021 to 28th January 2022).

IX, 05JAN-28JAN) Only including households, where the primary case tested positive after the holidays, 5th to 28th January 2022, rather than the full period (20th December 2021 to 28th January 2022), in order to exclude the atypical transmission patterns between Christmas 2021 and New Year's Eve 2021/22.

X, Excl. prev. HH infect.) Excluding all households with any previous infections, rather than just within the previous 60 days.

XI, Only Tested) Restricting the household contacts to those having been tested, rather than all household members.

XII, Only TestCenterDK) Only including households, where the primary case was identified by a sample from the community test track (TestCenter Denmark) to account for the potential sampling bias from the healthcare track (Section S1.2).

XIII, Control for Ct) Controlling for sample Ct value of the primary case using an additional explanatory variable, as differences in the viral load could potentially affect the results.

XIV, More vaccination groups) Splitting the "Fully vaccinated" category into four categories for both the primary case and household contacts.

The results from the 13 different specifications are qualitatively similar, which provide additional support the robustness of our conclusions.

In Table S17, we further provide unadjusted estimates for the infectiousness and susceptibility, i.e., excluding the control variables age, sex, household size and primary case sample date.

Lastly, to investigate the sensitivity of our results presented in Table 3, we also estimated our model by stratifying the sample (Table S18).

Table S12: Robustness I

**Model**	**I**		**II**		**III**		**IV**	
	**Main**		**14 day SAR**		**2-person households**		**Primary cases >10years**	
	**OR**	**(95%-CI)**	**OR**	**(95%-CI)**	**OR**	**(95%-CI)**	**OR**	**(95%-CI)**
**Household contact, vaccination status**								
Omicron BA.1 households								
Booster vaccinated	0.69	(0.64-0.75)	0.73	(0.67-0.78)	0.79	(0.66-0.94)	0.68	(0.62-0.74)
Fully vaccinated	ref	(.)	ref	(.)	ref	(.)	ref	(.)
Unvaccinated	1.27	(1.17-1.39)	1.33	(1.22-1.45)	1.13	(0.89-1.43)	1.23	(1.12-1.36)
Omicron BA.2 households								
Booster vaccinated	1.83	(1.57-2.14)	1.80	(1.54-2.10)	1.80	(1.22-2.65)	1.58	(1.29-1.93)
Fully vaccinated	2.26	(1.95-2.62)	2.24	(1.93-2.59)	1.93	(1.34-2.78)	1.93	(1.60-2.33)
Unvaccinated	2.54	(2.19-2.94)	2.45	(2.12-2.83)	2.29	(1.63-3.21)	2.12	(1.76-2.55)
**Primary case, vaccination status**								
Omicron BA.1 households								
Booster vaccinated	0.82	(0.75-0.91)	0.86	(0.78-0.94)	0.78	(0.65-0.94)	0.81	(0.73-0.90)
Fully vaccinated	ref	(.)	ref	(.)	ref	(.)	ref	(.)
Unvaccinated	0.98	(0.89-1.08)	0.98	(0.89-1.08)	0.86	(0.69-1.08)	0.98	(0.88-1.10)
Omicron BA.2 households								
Booster vaccinated	0.57	(0.48-0.68)	0.58	(0.49-0.69)	0.67	(0.45-0.99)	0.64	(0.52-0.78)
Fully vaccinated	0.66	(0.57-0.78)	0.63	(0.54-0.73)	0.79	(0.54-1.14)	0.76	(0.63-0.92)
Unvaccinated	ref	(.)	ref	(.)	ref	(.)	ref	(.)
**Primary case, age**								
0-9 years	2.19	(1.97-2.44)	2.27	(2.04-2.52)	1.43	(1.05-1.96)	0.92	(0.84-1.00)
10-19 years	0.94	(0.87-1.03)	1.05	(0.97-1.15)	0.68	(0.55-0.84)	1.67	(1.52-1.83)
20-29 years	ref	(.)	ref	(.)	ref	(.)	ref	(.)
30-39 years	1.71	(1.56-1.88)	1.67	(1.52-1.83)	0.99	(0.82-1.20)	1.77	(1.59-1.96)
40-49 years	1.82	(1.64-2.02)	1.78	(1.61-1.98)	1.52	(1.22-1.90)	1.81	(1.61-2.03)
50-59 years	1.86	(1.66-2.09)	1.85	(1.65-2.07)	1.78	(1.45-2.17)	2.26	(1.91-2.68)
60-69 years	2.39	(2.02-2.83)	2.26	(1.91-2.68)	1.99	(1.56-2.54)	3.11	(2.41-4.00)
70+ years	3.38	(2.62-4.35)	3.49	(2.70-4.51)	2.67	(1.95-3.67)	0.83	(0.75-0.93)
**Household contact, age**								
0-9 years	0.74	(0.67-0.81)	0.75	(0.68-0.82)	0.66	(0.49-0.89)	-	-
10-19 years	0.81	(0.74-0.88)	0.94	(0.87-1.02)	0.62	(0.48-0.80)	0.89	(0.81-0.98)
20-29 years	ref	(.)	ref	(.)	ref	(.)	ref	(.)
30-39 years	1.44	(1.33-1.57)	1.40	(1.28-1.52)	1.09	(0.90-1.32)	1.52	(1.38-1.67)
40-49 years	1.12	(1.03-1.22)	1.08	(0.99-1.17)	0.83	(0.67-1.02)	1.20	(1.09-1.32)
50-59 years	0.87	(0.80-0.96)	0.82	(0.75-0.90)	0.85	(0.70-1.04)	0.94	(0.85-1.04)
60-69 years	0.75	(0.65-0.86)	0.67	(0.59-0.77)	0.79	(0.62-1.00)	0.83	(0.72-0.96)
70+ years	0.61	(0.49-0.75)	0.55	(0.45-0.68)	0.69	(0.51-0.93)	0.68	(0.55-0.84)
**Household size**								
2 persons	1.15	(1.07-1.23)	1.05	(0.97-1.12)	-	-	1.20	(1.11-1.30)
3 persons	0.92	(0.86-0.98)	0.86	(0.81-0.92)	-	-	0.91	(0.85-0.98)
4 persons	ref	(.)	ref	(.)	-	-	ref	(.)
5 persons	0.90	(0.84-0.97)	0.95	(0.88-1.02)	-	-	0.92	(0.84-1.00)
6 persons	0.73	(0.64-0.84)	0.83	(0.72-0.94)	-	-	0.74	(0.63-0.87)
**Household contact, sex**								
Male	ref	ref	ref	(.)	ref	(.)	ref	(.)
Female	1.18	(1.14-1.22)	1.19	(1.15-1.24)	1.32	(1.18-1.48)	1.19	(1.14-1.24)
**Primary case, sex**								
Male	ref	(.)	ref	(.)	ref	(.)	ref	(.)
Female	0.98	(0.93-1.03)	0.99	(0.94-1.04)	0.98	(0.88-1.10)	1.00	(0.95-1.06)
**Primary case, sample date**								
Fixed effects - Not shown	-	-	-	-	-	-	-	-
Number of observations	50,588		50,588		7,762		41,667	
Number of households	22,678		22,678		7,762		19,537	

Notes: This table provides model estimates for the main specification (model I) as well as different robustness specifications. 95% confidence intervals are shown in parentheses. Standard errors are clustered on the household level.

Table S13: Robustness II

Model	I		V		VI		VII	
	Main		Only tested negative		Only cases on day 2-7		Only cases on day 3-7	
	OR	(95%-CI)	OR	(95%-CI)	OR	(95%-CI)	OR	(95%-CI)
**Household contact, vaccination status**								
Omicron BA.1 households								
Booster vaccinated	0.69	(0.64-0.75)	0.68	(0.61-0.76)	0.71	(0.65-0.77)	0.74	(0.68-0.81)
Fully vaccinated	ref	(.)	ref	(.)	ref	(.)	ref	(.)
Unvaccinated	1.27	(1.17-1.39)	1.48	(1.30-1.68)	1.30	(1.18-1.42)	1.28	(1.16-1.42)
Omicron BA.2 households								
Booster vaccinated	1.83	(1.57-2.14)	1.92	(1.52-2.41)	1.91	(1.63-2.25)	1.76	(1.48-2.10)
Fully vaccinated	2.26	(1.95-2.62)	2.31	(1.86-2.88)	2.26	(1.94-2.64)	2.00	(1.69-2.36)
Unvaccinated	2.54	(2.19-2.94)	3.44	(2.75-4.29)	2.51	(2.16-2.93)	2.19	(1.85-2.59)
**Primary case, vaccination status**								
Omicron BA.1 households								
Booster vaccinated	0.82	(0.75-0.91)	0.75	(0.65-0.85)	0.80	(0.72-0.89)	0.82	(0.73-0.92)
Fully vaccinated	ref	(.)	ref	(.)	ref	(.)	ref	(.)
Unvaccinated	0.98	(0.89-1.08)	1.02	(0.88-1.18)	0.98	(0.88-1.08)	0.97	(0.87-1.09)
Omicron BA.2 households								
Booster vaccinated	0.57	(0.48-0.68)	0.54	(0.41-0.69)	0.59	(0.49-0.70)	0.69	(0.57-0.85)
Fully vaccinated	0.66	(0.57-0.78)	0.66	(0.53-0.83)	0.67	(0.58-0.79)	0.75	(0.63-0.89)
Unvaccinated	ref	(.)	ref	(.)	ref	(.)	ref	(.)
**Primary case, age**								
0-9 years	2.19	(1.97-2.44)	2.00	(1.69-2.36)	2.34	(2.09-2.62)	2.46	(2.17-2.78)
10-19 years	0.94	(0.87-1.03)	0.83	(0.73-0.95)	1.02	(0.93-1.12)	1.07	(0.96-1.18)
20-29 years	ref	(.)	ref	(.)	ref	(.)	ref	(.)
30-39 years	1.71	(1.56-1.88)	1.55	(1.34-1.79)	1.70	(1.55-1.88)	1.68	(1.51-1.87)
40-49 years	1.82	(1.64-2.02)	1.78	(1.52-2.08)	1.79	(1.60-1.99)	1.77	(1.57-1.99)
50-59 years	1.86	(1.66-2.09)	1.87	(1.60-2.19)	1.80	(1.60-2.03)	1.79	(1.57-2.04)
60-69 years	2.39	(2.02-2.83)	2.23	(1.78-2.79)	2.29	(1.92-2.73)	2.25	(1.86-2.71)
70+ years	3.38	(2.62-4.35)	2.55	(1.81-3.59)	3.17	(2.44-4.13)	2.82	(2.14-3.72)
**Household contact, age**								
0-9 years	0.74	(0.67-0.81)	0.68	(0.59-0.79)	0.79	(0.71-0.87)	0.86	(0.77-0.96)
10-19 years	0.81	(0.74-0.88)	0.70	(0.62-0.80)	0.86	(0.78-0.94)	0.90	(0.81-0.99)
20-29 years	ref	(.)	ref	(.)	ref	(.)	ref	(.)
30-39 years	1.44	(1.33-1.57)	1.28	(1.13-1.45)	1.44	(1.32-1.58)	1.46	(1.32-1.61)
40-49 years	1.12	(1.03-1.22)	1.14	(1.00-1.29)	1.18	(1.08-1.29)	1.23	(1.12-1.36)
50-59 years	0.87	(0.80-0.96)	0.89	(0.78-1.02)	0.90	(0.81-0.99)	0.92	(0.83-1.03)
60-69 years	0.75	(0.65-0.86)	0.84	(0.68-1.02)	0.78	(0.68-0.91)	0.82	(0.70-0.96)
70+ years	0.61	(0.49-0.75)	0.86	(0.62-1.18)	0.63	(0.51-0.79)	0.68	(0.54-0.85)
**Household size**								
2 persons	1.15	(1.07-1.23)	1.29	(1.17-1.42)	1.14	(1.06-1.23)	1.11	(1.02-1.20)
3 persons	0.92	(0.86-0.98)	0.96	(0.87-1.05)	0.90	(0.85-0.97)	0.90	(0.84-0.97)
4 persons	ref	(.)	ref	(.)	ref	(.)	ref	(.)
5 persons	0.90	(0.84-0.97)	0.89	(0.79-1.00)	0.91	(0.85-0.99)	0.93	(0.86-1.01)
6 persons	0.73	(0.64-0.84)	0.82	(0.62-1.07)	0.73	(0.63-0.84)	0.76	(0.65-0.88)
**Household contact, sex**								
Male	ref	ref	ref	(.)	ref	(.)	ref	(.)
Female	1.18	(1.14-1.22)	1.16	(1.10-1.22)	1.17	(1.13-1.22)	1.15	(1.10-1.20)
**Primary case, sex**								
Male	ref	(.)	ref	(.)	ref	(.)	ref	(.)
Female	0.98	(0.93-1.03)	0.91	(0.85-0.98)	0.98	(0.93-1.03)	0.99	(0.94-1.05)
**Primary case, sample date**								
Fixed effects - Not shown	-	-	-	-	-	-	-	-
Number of observations	50,588		27,266		47,660		44,250	
Number of households	22,678		13,449		21,960		21,094	

Notes: This table provides model estimates for the main specification (model I) as well as different robustness specifications, i.e., adjusted regression estimates. 95%-confidence intervals are shown in parentheses with cluster-robust standard errors at the household level.

Table S14: Robustness III

Model	I		VIII		IX		X	
	Main		20DEC-04JAN		05JAN-28JAN		Excl. prev. HH infect.	
	**OR**	**(95%-CI)**	**OR**	**(95%-CI)**	**OR**	**(95%-CI)**	**OR**	**(95%-CI)**
**Household contact, vaccination status**								
Omicron BA.1 households								
Booster vaccinated	0.69	(0.64-0.75)	0.69	(0.61-0.80)	0.70	(0.63-0.77)	0.62	(0.56-0.67)
Fully vaccinated	ref	(.)	ref	(.)	ref	(.)	ref	(.)
Unvaccinated	1.27	(1.17-1.39)	1.22	(1.06-1.41)	1.31	(1.17-1.47)	1.07	(0.97-1.18)
Omicron BA.2 households								
Booster vaccinated	1.83	(1.57-2.14)	1.59	(0.95-2.64)	1.82	(1.53-2.16)	1.51	(1.26-1.80)
Fully vaccinated	2.26	(1.95-2.62)	2.15	(1.34-3.45)	2.27	(1.93-2.67)	2.16	(1.82-2.55)
Unvaccinated	2.54	(2.19-2.94)	2.42	(1.52-3.86)	2.56	(2.17-3.01)	1.91	(1.62-2.26)
**Primary case, vaccination status**								
Omicron BA.1 households								
Booster vaccinated	0.82	(0.75-0.91)	0.75	(0.64-0.87)	0.87	(0.77-1.00)	0.76	(0.69-0.85)
Fully vaccinated	ref	(.)	ref	(.)	ref	(.)	ref	(.)
Unvaccinated	0.98	(0.89-1.08)	0.89	(0.75-1.06)	1.05	(0.93-1.19)	0.95	(0.85-1.06)
Omicron BA.2 households								
Booster vaccinated	0.57	(0.48-0.68)	0.53	(0.31-0.93)	0.64	(0.52-0.77)	0.61	(0.50-0.74)
Fully vaccinated	0.66	(0.57-0.78)	0.64	(0.39-1.05)	0.70	(0.59-0.83)	0.73	(0.61-0.87)
Unvaccinated	ref	(.)	ref	(.)	ref	(.)	ref	(.)
**Primary case, age**								
0-9 years	2.19	(1.97-2.44)	2.50	(1.89-3.31)	2.04	(1.81-2.30)	2.16	(1.91-2.43)
10-19 years	0.94	(0.87-1.03)	0.94	(0.79-1.12)	0.91	(0.82-1.01)	0.86	(0.78-0.95)
20-29 years	ref	(.)	ref	(.)	ref	(.)	ref	(.)
30-39 years	1.71	(1.56-1.88)	2.01	(1.71-2.37)	1.58	(1.41-1.77)	1.60	(1.45-1.77)
40-49 years	1.82	(1.64-2.02)	2.57	(2.15-3.06)	1.50	(1.32-1.71)	1.81	(1.62-2.03)
50-59 years	1.86	(1.66-2.09)	2.20	(1.81-2.69)	1.68	(1.46-1.93)	1.85	(1.63-2.09)
60-69 years	2.39	(2.02-2.83)	3.09	(2.34-4.08)	2.06	(1.67-2.55)	2.42	(2.02-2.90)
70+ years	3.38	(2.62-4.35)	3.91	(2.54-6.02)	3.11	(2.27-4.27)	3.35	(2.57-4.38)
**Household contact, age**								
0-9 years	0.74	(0.67-0.81)	0.66	(0.55-0.80)	0.77	(0.69-0.87)	0.76	(0.69-0.85)
10-19 years	0.81	(0.74-0.88)	0.69	(0.59-0.81)	0.87	(0.78-0.96)	0.76	(0.69-0.83)
20-29 years	ref	(.)	ref	(.)	ref	(.)	ref	(.)
30-39 years	1.44	(1.33-1.57)	1.21	(1.02-1.43)	1.55	(1.40-1.70)	1.38	(1.26-1.51)
40-49 years	1.12	(1.03-1.22)	1.01	(0.85-1.19)	1.19	(1.08-1.31)	1.10	(1.00-1.21)
50-59 years	0.87	(0.80-0.96)	0.76	(0.63-0.90)	0.95	(0.85-1.06)	0.86	(0.78-0.96)
60-69 years	0.75	(0.65-0.86)	0.66	(0.51-0.85)	0.81	(0.69-0.96)	0.72	(0.62-0.84)
70+ years	0.61	(0.49-0.75)	0.51	(0.35-0.75)	0.67	(0.52-0.86)	0.58	(0.46-0.72)
**Household size**								
2 persons	1.15	(1.07-1.23)	1.20	(1.04-1.37)	1.12	(1.02-1.22)	1.08	(1.00-1.17)
3 persons	0.92	(0.86-0.98)	0.90	(0.79-1.03)	0.93	(0.86-1.00)	0.90	(0.84-0.97)
4 persons	ref	(.)	ref	(.)	ref	(.)	ref	(.)
5 persons	0.90	(0.84-0.97)	0.83	(0.70-0.98)	0.93	(0.85-1.00)	0.93	(0.85-1.01)
6 persons	0.73	(0.64-0.84)	0.76	(0.57-1.01)	0.72	(0.62-0.84)	0.73	(0.62-0.85)
**Household contact, sex**								
Male	ref	ref	ref	(.)	ref	(.)	ref	(.)
Female	1.18	(1.14-1.22)	1.15	(1.07-1.25)	1.19	(1.14-1.24)	1.19	(1.14-1.23)
**Primary case, sex**								
Male	ref	(.)	ref	(.)	ref	(.)	ref	(.)
Female	0.98	(0.93-1.03)	1.05	(0.95-1.16)	0.96	(0.91-1.02)	0.98	(0.93-1.04)
**Primary case, sample date**								
Fixed effects - Not shown	-	-	-	-	-	-	-	-
Number of observations	50,588		12,451		38,137		40,835	
Number of households	22,678		6,110		16,568		18,836	

Notes: This table provides model estimates for the main specification (model I) as well as different robustness specifications, i.e., adjusted regression estimates. 95%-confidence intervals are shown in parentheses with cluster-robust standard errors at the household level.

Table S15: Robustness IV

Model	I		XI		XII		XIII	
	Main		Only Tested		Only TestCenterDK		Control for Ct	
	OR	(95%-CI)	OR	(95%-CI)	OR	(95%-CI)	OR	(95%-CI)
**Household contact, vaccination status**								
Omicron BA.1 households								
Booster vaccinated	0.69	(0.64-0.75)	0.62	(0.57-0.67)	0.66	(0.61-0.72)	0.66	(0.60-0.72)
Fully vaccinated	ref	(.)	ref	(.)	ref	(.)	ref	(.)
Unvaccinated	1.27	(1.17-1.39)	1.58	(1.44-1.73)	1.29	(1.17-1.42)	1.29	(1.17-1.43)
Omicron BA.2 households								
Booster vaccinated	1.83	(1.57-2.14)	2.08	(1.75-2.46)	1.79	(1.50-2.13)	1.68	(1.41-2.00)
Fully vaccinated	2.26	(1.95-2.62)	3.01	(2.56-3.54)	2.19	(1.86-2.59)	2.09	(1.77-2.47)
Unvaccinated	2.54	(2.19-2.94)	4.23	(3.60-4.97)	2.56	(2.16-3.02)	2.42	(2.05-2.86)
**Primary case, vaccination status**								
Omicron BA.1 households								
Booster vaccinated	0.82	(0.75-0.91)	0.72	(0.65-0.80)	0.81	(0.72-0.91)	0.84	(0.75-0.95)
Fully vaccinated	ref	(.)	ref	(.)	ref	(.)	ref	(.)
Unvaccinated	0.98	(0.89-1.08)	1.05	(0.94-1.16)	0.95	(0.85-1.06)	0.93	(0.83-1.04)
Omicron BA.2 households								
Booster vaccinated	0.57	(0.48-0.68)	0.40	(0.33-0.48)	0.56	(0.46-0.69)	0.60	(0.49-0.74)
Fully vaccinated	0.66	(0.57-0.78)	0.54	(0.46-0.64)	0.67	(0.56-0.80)	0.70	(0.59-0.83)
Unvaccinated	ref	(.)	ref	(.)	ref	(.)	ref	(.)
**Primary case, age**								
0-9 years	2.19	(1.97-2.44)	1.48	(1.31-1.66)	2.11	(1.87-2.39)	2.19	(1.93-2.47)
10-19 years	0.94	(0.87-1.03)	0.71	(0.64-0.78)	0.94	(0.85-1.03)	0.95	(0.86-1.04)
20-29 years	ref	(.)	ref	(.)	ref	(.)	ref	(.)
30-39 years	1.71	(1.56-1.88)	1.58	(1.43-1.76)	1.70	(1.53-1.89)	1.69	(1.52-1.87)
40-49 years	1.82	(1.64-2.02)	1.61	(1.44-1.80)	1.77	(1.58-1.99)	1.75	(1.56-1.97)
50-59 years	1.86	(1.66-2.09)	1.76	(1.56-2.00)	1.82	(1.60-2.07)	1.77	(1.56-2.02)
60-69 years	2.39	(2.02-2.83)	2.21	(1.83-2.65)	2.47	(2.03-3.02)	2.37	(1.94-2.90)
70+ years	3.38	(2.62-4.35)	2.87	(2.12-3.88)	3.93	(2.83-5.44)	3.59	(2.57-5.01)
**Household contact, age**								
0-9 years	0.74	(0.67-0.81)	0.69	(0.62-0.77)	0.75	(0.67-0.83)	0.75	(0.67-0.83)
10-19 years	0.81	(0.74-0.88)	0.69	(0.63-0.76)	0.85	(0.77-0.93)	0.84	(0.76-0.93)
20-29 years	ref	(.)	ref	(.)	ref	(.)	ref	(.)
30-39 years	1.44	(1.33-1.57)	1.39	(1.27-1.53)	1.45	(1.32-1.60)	1.46	(1.32-1.61)
40-49 years	1.12	(1.03-1.22)	1.10	(1.00-1.21)	1.17	(1.06-1.28)	1.17	(1.06-1.29)
50-59 years	0.87	(0.80-0.96)	0.90	(0.82-1.00)	0.91	(0.82-1.01)	0.91	(0.82-1.01)
60-69 years	0.75	(0.65-0.86)	0.91	(0.77-1.07)	0.75	(0.64-0.88)	0.74	(0.63-0.87)
70+ years	0.61	(0.49-0.75)	0.97	(0.74-1.27)	0.64	(0.50-0.82)	0.64	(0.50-0.83)
**Household size**								
2 persons	1.15	(1.07-1.23)	1.09	(1.01-1.18)	1.18	(1.09-1.28)	1.19	(1.10-1.29)
3 persons	0.92	(0.86-0.98)	0.99	(0.92-1.05)	0.92	(0.86-0.99)	0.93	(0.87-1.00)
4 persons	ref	(.)	ref	(.)	ref	(.)	ref	(.)
5 persons	0.90	(0.84-0.97)	0.94	(0.87-1.02)	0.93	(0.86-1.01)	0.93	(0.85-1.00)
6 persons	0.73	(0.64-0.84)	0.85	(0.74-0.99)	0.75	(0.64-0.87)	0.74	(0.64-0.86)
**Household contact, sex**								
Male	ref	ref	ref	(.)	ref	(.)	ref	(.)
Female	1.18	(1.14-1.22)	1.11	(1.07-1.16)	1.18	(1.14-1.23)	1.18	(1.13-1.23)
**Primary case, sex**								
Male	ref	(.)	ref	(.)	ref	(.)	ref	(.)
Female	0.98	(0.93-1.03)	0.94	(0.89-0.99)	0.96	(0.91-1.02)	0.97	(0.91-1.02)
**Primary case, sample date**								
Fixed effects - Not shown	-	-	-	-	-	-	-	-
**Ct value**								
16-18	-	-	-	-	-	-	2.09	(1.29-3.40)
18-20	-	-	-	-	-	-	1.52	(1.27-1.82)
20-22	-	-	-	-	-	-	1.55	(1.38-1.74)
22-24	-	-	-	-	-	-	1.35	(1.23-1.49)
24-26	-	-	-	-	-	-	1.14	(1.04-1.25)
26-28	-	-	-	-	-	-	1.02	(0.93-1.13)
28-30	-	-	-	-	-	-	ref	(.)
30-32	-	-	-	-	-	-	0.90	(0.80-1.00)
32-34	-	-	-	-	-	-	0.85	(0.75-0.96)
34-36	-	-	-	-	-	-	0.90	(0.71-1.13)
36-38	-	-	-	-	-	-	0.78	(0.27-2.25)
Number of observations	50,588		42,201		40,643		40,551	
Number of households	22,678		20,669		18,047		17,987	

Notes: This table provides model estimates for the main specification (model I) as well as different robustness specifications, i.e., adjusted regression estimates. 95%-confidence intervals are shown in parentheses with cluster-robust standard errors at the household level.

Table S16: Robustness V

Model	XIV	
	More vaccination groups	
	OR	(95%-CI)
**Contact vaccination status**		
Omicron BA.1 households		
Booster vaccinated	0.61	(0.56-0.66)
Fully vaccinated & previous infection	0.43	(0.35-0.53)
Previous infection (no vaccination)	0.49	(0.40-0.61)
Fully vaccinated (no previous infection)	ref	(.)
Unvaccinated	1.06	(0.97-1.16)
Omicron BA.2 households		
Booster vaccinated	0.56	(0.35-0.91)
Fully vaccinated & previous infection	0.32	(0.19-0.52)
Previous infection (no vaccination)	0.43	(0.26-0.71)
Fully vaccinated (no previous infection)	0.82	(0.51-1.33)
Unvaccinated	0.74	(0.45-1.20)
**Primary case vaccination status**		
Omicron BA.1 households		
Booster vaccinated	0.78	(0.70-0.86)
Fully vaccinated & previous infection	0.58	(0.46-0.74)
Previous infection (no vaccination)	0.76	(0.59-0.97)
Fully vaccinated (no previous infection)	ref	(.)
Unvaccinated	0.93	(0.84-1.03)
Omicron BA.2 households		
Booster vaccinated	1.53	(0.94-2.49)
Fully vaccinated & previous infection	ref	(.)
Previous infection (no vaccination)	1.51	(0.89-2.58)
Fully vaccinated (no previous infection)	1.92	(1.18-3.12)
Unvaccinated	2.12	(1.29-3.46)
**Primary case, age**		
0-9 years	2.20	(1.97-2.45)
10-19 years	0.92	(0.84-1.00)
20-29 years	ref	(.)
30-39 years	1.69	(1.54-1.85)
40-49 years	1.82	(1.64-2.02)
50-59 years	1.87	(1.67-2.10)
60-69 years	2.39	(2.02-2.83)
70+ years	3.40	(2.64-4.39)
**Household contact, age**		
0-9 years	0.80	(0.72-0.88)
10-19 years	0.79	(0.73-0.86)
20-29 years	ref	(.)
30-39 years	1.42	(1.31-1.55)
40-49 years	1.12	(1.03-1.22)
50-59 years	0.88	(0.80-0.96)
60-69 years	0.75	(0.65-0.86)
70+ years	0.60	(0.49-0.74)
**Household size**		
2 persons	1.15	(1.07-1.23)
3 persons	0.92	(0.86-0.98)
4 persons	ref	(.)
5 persons	0.92	(0.85-0.99)
6 persons	0.76	(0.66-0.87)
**Household contact, sex**		
Male	ref	(.)
Female	1.19	(1.14-1.23)
**Primary case, sex**		
Male	ref	(.)
Female	0.98	(0.94-1.03)
**Primary case, sample date**		
Fixed effects - Not shown	-	-
Number of observations	50,588	
Number of households	22,678	

Notes: This table provides model estimates for the main specification with more vaccination groups, i.e., splitting the "Fully vaccinated" category into four categories both for the primary case and household contacts. 95%-confidence intervals are shown in parentheses with cluster-robust standard errors at the household level.

Table S17: Robustness VI

Model	XV Unadjusted		I Main	
	OR	(95%-CI)	OR	(95%-CI)
**Household contact, vaccination status**				
Omicron BA.1 households				
Booster vaccinated	0.68	(0.63-0.73)	0.69	(0.64-0.75)
Fully vaccinated	ref	(.)	ref	(.)
Unvaccinated	1.05	(0.98-1.13)	1.27	(1.17-1.39)
Omicron BA.2 households				
Booster vaccinated	2.76	(2.45-3.11)	1.83	(1.57-2.14)
Fully vaccinated	3.37	(3.00-3.79)	2.26	(1.95-2.62)
Unvaccinated	3.07	(2.74-3.44)	2.54	(2.19-2.94)
**Primary case, vaccination status**				
Omicron BA.1 households				
Booster vaccinated	1.08	(0.99-1.18)	0.82	(0.75-0.91)
Fully vaccinated	ref	(.)	ref	(.)
Unvaccinated	1.41	(1.29-1.53)	0.98	(0.89-1.08)
Omicron BA.2 households				
Booster vaccinated	0.45	(0.39-0.52)	0.57	(0.48-0.68)
Fully vaccinated	0.39	(0.35-0.45)	0.66	(0.57-0.78)
Unvaccinated	ref	(.)	ref	(.)
**Primary case, age**				
0-9 years	-	-	2.19	(1.97-2.44)
10-19 years	-	-	0.94	(0.87-1.03)
20-29 years	-	-	ref	(.)
30-39 years	-	-	1.71	(1.56-1.88)
40-49 years	-	-	1.82	(1.64-2.02)
50-59 years	-	-	1.86	(1.66-2.09)
60-69 years	-	-	2.39	(2.02-2.83)
70+ years	-	-	3.38	(2.62-4.35)
**Household contact, age**				
0-9 years	-	-	0.74	(0.67-0.81)
10-19 years	-	-	0.81	(0.74-0.88)
20-29 years	-	-	ref	(.)
30-39 years	-	-	1.44	(1.33-1.57)
40-49 years	-	-	1.12	(1.03-1.22)
50-59 years	-	-	0.87	(0.80-0.96)
60-69 years	-	-	0.75	(0.65-0.86)
70+ years	-	-	0.61	(0.49-0.75)
**Household size**				
2 persons	-	-	1.15	(1.07-1.23)
3 persons	-	-	0.92	(0.86-0.98)
4 persons	-	-	ref	(.)
5 persons	-	-	0.90	(0.84-0.97)
6 persons	-	-	0.73	(0.64-0.84)
**Household contact, sex**				
Male	-	-	ref	ref
Female	-	-	1.18	(1.14-1.22)
**Primary case, sex**				
Male	-	-	ref	(.)
Female	-	-	0.98	(0.93-1.03)
**Primary case, sample date**				
Fixed effects - Not shown	Not included		Included	
Number of observations	50,588		50,588	
Number of households	22,678		22,678	

Notes: This table provides model estimates for the unadjusted main specification This table provides model estimates for the main specification (model I) as well as unadjusted estimates, i.e., excluding the control variables age, sex household size and primary case sample date. 95%-confidence intervals are shown in parentheses with cluster-robust standard errors at the household level.

Table S18: Relative effect of Omicron BA.2 vs. BA.1, by stratification

	**Susceptibility (Household contacts)**			**Infectiousness (Primary case)**		
	**Unvaccinated**	**Fully vaccinated**	**Booster vaccinated**	**Unvaccinated**	**Fully vaccinated**	**Booster vaccinated**
**Omicron BA.2 households**	2.50	2.07	2.50	3.53	1.55	1.61
	(2.05-3.05)	(1.66-2.58)	(1.98-3.16)	(2.9-4.29)	(1.26-1.89)	(1.19-2.18)
**Omicron BA.1 households**	ref	ref	ref	ref	ref	ref
	(.)	(.)	(.)	(.)	(.)	(.)
Number of observations	13,520	17,433	19,635	14,813	24,379	11,396
Number of households	9,050	12,406	13,502	5,782	10,511	6,385

Notes: This table provides model estimates similar to Table 3, but with stratification of the sample. For the susceptibility estimates, we stratify by the vaccination status of the household contacts. For the infectiousness estimates, we stratify by the vaccination status of the primary case. 95%-confidence intervals are shown in parentheses with cluster-robust standard errors at the household level.
