# Supplementary material for: Household transmission of SARS-CoV-2 Omicron variant of concern subvariants BA.1 and BA.2 in Denmark
Source: Nat Commun. 2022 Sep 30;13:5760. doi: 10.1038/s41467-022-33498-0 (PMC9524324; doi:10.1038/s41467-022-33498-0)
Supplement: Supplementary file 3 — Reporting Summary [file 41467_2022_33498_MOESM3_ESM.pdf]

nature portfolio

Corresponding author(s): Frederik Plesner Lyngse

Last updated by author(s): Sep 2, 2022

Reporting Summary

Nature Portfolio wishes to improve the reproducibility of the work that we publish. This form provides structure for consistency and transparency in reporting. For further information on Nature Portfolio policies, see our Editorial Policies and the Editorial Policy Checklist.

Statistics

For all statistical analyses, confirm that the following items are present in the figure legend, table legend, main text, or Methods section.

n/a	Confirmed	
☐	☒	The exact sample size (*n*) for each experimental group/condition, given as a discrete number and unit of measurement
☐	☒	A statement on whether measurements were taken from distinct samples or whether the same sample was measured repeatedly
☒	☐	The statistical test(s) used AND whether they are one- or two-sided<br>*Only common tests should be described solely by name; describe more complex techniques in the Methods section.*
☐	☒	A description of all covariates tested
☐	☒	A description of any assumptions or corrections, such as tests of normality and adjustment for multiple comparisons
☐	☒	A full description of the statistical parameters including central tendency (e.g. means) or other basic estimates (e.g. regression coefficient) AND variation (e.g. standard deviation) or associated estimates of uncertainty (e.g. confidence intervals)
☐	☒	For null hypothesis testing, the test statistic (e.g. *F*, *t*, *r*) with confidence intervals, effect sizes, degrees of freedom and *P* value noted<br>*Give P values as exact values whenever suitable.*
☒	☐	For Bayesian analysis, information on the choice of priors and Markov chain Monte Carlo settings
☒	☐	For hierarchical and complex designs, identification of the appropriate level for tests and full reporting of outcomes
☒	☐	Estimates of effect sizes (e.g. Cohen's *d*, Pearson's *r*), indicating how they were calculated

Our web collection on statistics for biologists contains articles on many of the points above.

Software and code

Policy information about availability of computer code

Data collection	We performed no data collection or sequencing specifically for this study. We did not use any software for data collection.<br>Denmark performs testing and analysis of tests as part of the public response against the SARS-CoV-2 epidemic. The Danish COVID-19 Genome Consortium performs whole-genome sequencing of positive RT-PCR tests as part of the response. Variants were called using Pangolin (version 04.00.06).
Data analysis	We used SAS 9.4 to manage and analyze the data. The code used for this study can be downloaded from a public repository: https://github.com/Flyngse/SARS-CoV-2_Omicron_BA1_BA2

For manuscripts utilizing custom algorithms or software that are central to the research but not yet described in published literature, software must be made available to editors and reviewers. We strongly encourage code deposition in a community repository (e.g. GitHub). See the Nature Portfolio guidelines for submitting code & software for further information.

Data

Policy information about availability of data

All manuscripts must include a data availability statement. This statement should provide the following information, where applicable:

- Accession codes, unique identifiers, or web links for publicly available datasets
- A description of any restrictions on data availability
- For clinical datasets or third party data, please ensure that the statement adheres to our policy

The data used in this study are available under restricted access due to Danish data protection legislation. The data are available for research upon request to The Danish Health Data Authority and Statens Serum Institut and within the framework of the Danish data protection legislation and any required permission from Authorities. We performed no data collection or sequencing specifically for this study. Consensus genome data from the Danish cases are routinely shared publicly

at GISAID (www.gisaid.org). We used the following Danish administrative registers: Central Person Register (CPR), Danish Microbiology Database (MiBa), Danish Vaccination Register (DDV), and data set on variants for positive RT-PCR tests.

Field-specific reporting

Please select the one below that is the best fit for your research. If you are not sure, read the appropriate sections before making your selection.

☒ Life sciences ☐ Behavioural & social sciences ☐ Ecological, evolutionary & environmental sciences

For a reference copy of the document with all sections, see nature.com/documents/nr-reporting-summary-flat.pdf

Life sciences study design

All studies must disclose on these points even when the disclosure is negative.

Sample size	No predetermined sample size calculation was performed. Our data include the full population of Denmark. We used data on primary cases from 20th December 2021 to 28th January 2022, and allowed a 7-day follow-up period for household contacts, i.e., until 4th February 2022. A primary case was defined as the first individual in a household testing positive with an RT-PCR test within the study period and being identified with the Omicron VOC BA.1 or BA.2 by whole genome sequencing (WGS). We followed all tests of other household contacts in the follow-up period. These restrictions leaves us with a sample of 22,678 primary cases and 17,319 secondary infections among 50,588 household contacts, which should be a sufficient large sample for the purpose of our study.
Data exclusions	We exclude households with less than one and more than six members. We exclude households with a positive RT-PCR test within the previous 60 days.
Replication	The code used for this study can be downloaded from a public repository: https://github.com/Flyngse/SARS-CoV-2_Omicron_BA1_BA2. The analysis can be replicated with data access through the Statens Serum Institut and Danish Health Data Authority.
Randomization	Randomization was not applicable, as the study uses observational data from administrative registers.
Blinding	Blinding was not applicable, as the study uses observational data from administrative registers.

Reporting for specific materials, systems and methods

We require information from authors about some types of materials, experimental systems and methods used in many studies. Here, indicate whether each material, system or method listed is relevant to your study. If you are not sure if a list item applies to your research, read the appropriate section before selecting a response.

Materials & experimental systems

n/a	Involved in the study	
☒	☐	Antibodies
☒	☐	Eukaryotic cell lines
☒	☐	Palaeontology and archaeology
☒	☐	Animals and other organisms
☒	☐	Human research participants
☒	☐	Clinical data
☒	☐	Dual use research of concern

Methods

n/a	Involved in the study	
☒	☐	ChIP-seq
☒	☐	Flow cytometry
☒	☐	MRI-based neuroimaging
